# Structure analysis of yeast glutaredoxin Grx6 protein produced in *Escherichia coli*

**DOI:** 10.1186/s41021-018-0103-6

**Published:** 2018-08-06

**Authors:** Mohnad Abdalla, Wafa Ali Eltayb, Amr Ahmed El-Arabey, Raihan Mo, T. I. M. Dafaalla, Hamed I. Hamouda, Eijaz Ahmed Bhat, Annoor Awadasseid, Hassan Abdellha Ahmed Ali

**Affiliations:** 1 Faculty of Science and Technology, Omdurman Islamic University, Khartoum, Sudan; 2 School of Life Sciences, University of Science and Technology of China, Hefei, Anhui 230027 People’s Republic of China; 3 Qingdao Institute of Bioenergy and Bioprocess Technology, Qingdao Shi, Shandong Sheng 266000 People’s Republic of China; 4 Faculty of Science and Technology, Shendi University, Shendi, Nher Anile Sudan; 5 College of Education, Sinnar University, 11147 Sinnar, Sudan; 6 School of Biotechnology and Graduate School of Biochemistry, Yeungnam, 280, Daehak-ro, Gyeongsan-si, Gyeongsangbuk-do 712-749 South Korea; 7 Department of Biochemistry and Molecular Biology, Dalian Medical University, Dalian, 116044 China; 8 Faculty of Medicine, Nile Valley University, Atbara, Sudan

**Keywords:** Grx6, Purification, *Saccharomyces cerevisiae*, Fe-S, Crystallization

## Abstract

**Background:**

Grx6 is a yeast Golgi/endoplasmic reticulum protein involved in iron-sulfur binding that belongs to monothiol glutaredoxin-protein family. Grx6 has been biochemically characterized previously. Grx6 contains a conserved cysteine residue (Cys-136). Depending on the active-site sequences, Grxs can be classified to classic dithiol Grxs with a CXXC motif known as classes II and monothiol Grxs with a CXXS motif known as classes I, and Grx6 belongs to the class I with a CSYS motif.

**Results:**

Our results showed how the loop between the N-terminal and C-terminal can affect the stability. When Grx6 was incubated with FeSO_4_·7H_2_O and (NH_4_)_2_Fe(SO_4_)_2_·6H_2_O, a disulfide bond was formed between the cysteine 136 and glutathione, and the concentration of dimer and tetramer was increased. The results presented various levels of stability of Grx6 with mutant and deleted amino acids. We also highlighted the difference between the monomer and dimer forms of the Grx6, in addition to comparison of the Fe-S cluster positions among holo forms of poplar Grx-C1, human Grx2 and *Saccharomyces cerevisiae* Grx6.

**Conclusions:**

In this paper, we used a combination of spectroscopic and proteomic techniques to analyse the sequence and to determine the affected mutations and deletions in the stability of Grx6. Our results have increased the knowledge about the differences between monomer and dimer structures in cellular processes and proteins whose roles and functions depend on YCA1 in yeast.

**Electronic supplementary material:**

The online version of this article (10.1186/s41021-018-0103-6) contains supplementary material, which is available to authorized users.

## Background

Glutaredoxin proteins (Grx) have thiol reductase activity. Glutaredoxins are necessary to reduce glutathione (GSH) as the electron donor [1]. Now, the assumptive glutaredoxin sequences consist of an active site that contains one or two conserved cysteines together with two non-conserved residues among them. Previous studies have shown that two conserved cysteines are necessary for reducing protein disulfides. In addition, only the cysteine locus at the N-terminal is essential for the reduction of incorporated disulfides with glutathione. To date, in *Saccharomyces cerevisiae*, ten various glutaredoxins have been found. Eight of them have been biochemically characterized, and three of five dimensions are available in the PDB database [2–4].

In addition, Grx6 regulates the glutathionylation of thiols of Golgi/endoplasmic reticulum target proteins, and as a result, it regulates the equilibrium between oxidized and reduced glutathione in the lumen of these compartments. One of the main functions of glutathionylation is to protect the cell against oxidative stress. Deletion of Grx5 resulted in serious growth defects by inducing the accumulation of the iron in the cell. Some mutations in Grx6, but not all, have deficient phenotypes in intracellular calcium transporters, such as the Golgi Pmr1 protein. Grx6 is involved in several biological processes and found in several cellular components (Table 1). These results show that any mutation in Grx6 causes defects in the pathways of the cells. Interestingly, mutagenizing the active site Cys-136 codon of the GRX6 open-reading frame (ORF) into a Ser codon makes reduction in the level of Ca^2+^ in the ER, whereas Ca^2+^ accumulation occurs in the cytosol. Fiddling mutants in Grx6 show a more intense unfolded protein response compared to wild-type cells upon treatment [5].

**Table 1:** Grx6 Gene Ontology

Molecular function	1. 2 iron, 2 sulfur cluster binding 2. Electron carrier activity 3. Glutathione-disulfide reductase activity 4. Iron ion binding 5. Metal ion binding 6. Protein disulfide oxidoreductase activity 7. Protein homodimerization activity
Biological process	1. Cell redox homeostasis 2. Cellular oxidant detoxification 3. Cellular response to oxidative stress 4. Oxidation-reduction process
Cellular component	1. Golgi apparatus 2. Golgi lumen 3. Cis-Golgi network 4. Endoplasmic reticulum membrane 5. Fungal-type vacuole 6. Integral component of membrane 7. Vacuole

In this work, we address a three-dimensional model of Grx6N-domain presented based on the known structure of different glutaredoxins. The Grx6N-domain shows a non-classic thioredoxin fold structure. Grx6N-domain is involved in the formation of a Grx6C domain cleft. However, Grx6N-domain sequences do not contain a cysteine residue. In addition, our studies have shown that the Grx6 cysteine locus at the C-terminal is essential for the biological activity of the Grx6 protein. Site-directed mutagenesis studies indicate that some residues in the N-domain can affect the expression of the whole protein. Despite these observations, the specific role of N-domain in the formation of a Grx6C domain is still not clear.

## Methods

### Cloning, expression and purification of *S. cerevisiae* Grx6 in *E. coli*

To investigate the number of Fe/S clusters in Grx6 in more detail, the coding regions of Grx6/YDL010W (without the signal peptide) were PCR-amplified from *S. cerevisiae* S288c genomic DNA and cloned into the pET22b(+) vector (Novagen, Cambridge) between the NdeI and XhoI sites. Extra sequences encoding residues (LEHHHHHH) were added at the C-terminus. These plasmids were transformed into *E. coli* strain BL21 (Biolabs, Massachusetts) by heat shock and induced using 0.02 mM IPTG at 16 °C for ~ 20 h after the OD_800_ nm reached 0.8. The cells were collected by centrifugation and resuspended in buffer (20 mM NaCl, 50 mM Tris–HCl, pH 8.0). After 20 min of sonication, which was followed by centrifugation at 30,000 xg for 25 min, the supernatant was pooled and loaded onto a Ni-NTA column (GE Healthcare, Chalfont). The target protein was eluted with 250 mM imidazole and loaded onto a Superdex 75 column preequilibrated.

### Site-directed mutagenesis

The site-directed mutagenesis primer was designed (Additional file 1: Table S1), and mutations were introduced into Grx6 by PCR. The plasmid was purified using an AXYGEN™ kit (Axygen Scientific, Beijing), and then the plasmid was cut by Dpn1 (TAKARA, Beijing) using 1 μl from the enzyme, 4 μl buffer B and a total volume up to 50 μl, which was incubated overnight at 37 °C. This was purified again using an AXYGE kit, and then the plasmid was linked using a CE II enzyme VAZYME (Vazyme Biotech, Nanjing) with 2 μl and 4 μl from the buffer and a total volume up to 20 μl, which was incubated in ice for 30 min. The final reaction was transformed into competent cells. Point and deletion mutations were confirmed by DNA sequencing.

### Nature of the Fe–S Centre in Grx6

The nature of the Fe–S centrein dimer Grx6 was assessed by using a spectroscopic technique. The sample purified to a dimer contained 0.1 mol of Fe per mol of protein.

### Crystallization

Grx6 crystals were grown at 14 °C using the sitting drop vapour diffusion method. The dimer, using 1.0 μl protein sample, was prepared as described above with an equal volume of reservoir solution (0.1 M HEPES sodium pH 7.5 and 0.8 M potassium sodium tartrate tetrahydrate). The monomer crystals were grown at 14 °C by the hanging drop method with the following condition: salt: 0.2 M magnesium chloride hexahydrate; buffer: 0.1 M tris-hydrochloride pH 8.5; and precipitant: 30% *w*/*v* polyethylene glycol 4000.

### Sequence analysis, protein–ligand interaction and modelling

The disorder profile, hydrophilicity and hydrophobicity profiles were analysed using the ExPASy [6], while protein–ligand interaction was created from the Grx6 PDB file [2], and the Grx6 N domain model was built by using the Phyre2 [7].

## Results and discussion

### Gel filtration

**Fig. 1:**
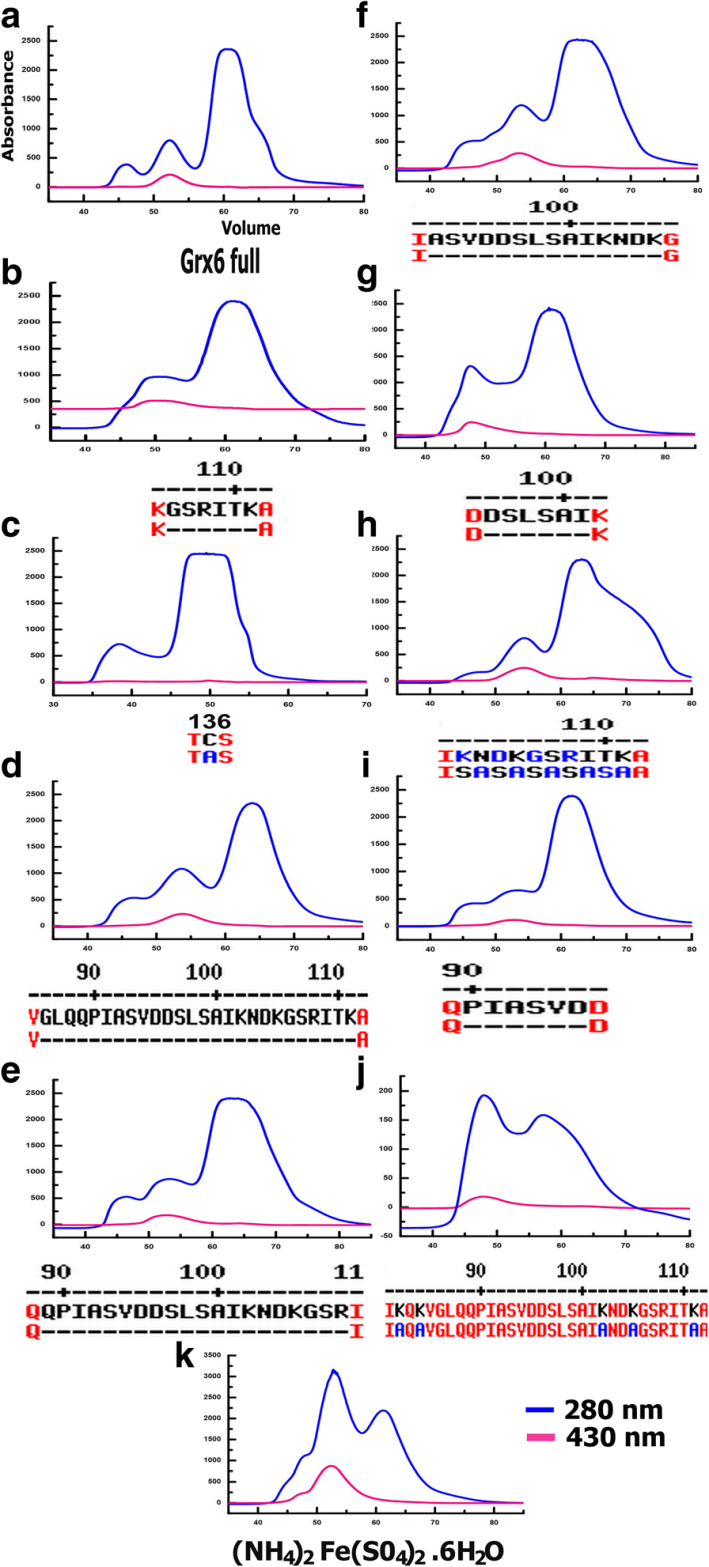
Gel filtration test profiles of freshly purified recombinant ScGrx6 with various mutations and deletions in the sequence. Chromatogram shows the elution profiles of 280 nm (blue) and 430 nm (pink) with size-exclusion chromatography on the Superdex 75 column

Grxs have a common active site motif CxxC/S located at a surface loop that is accessible to GSH. Gel-filtration chromatography showed that freshly purified full lengths of Grx6 have three successive peaks: the first one has a higher oligomerization state, the second has a brown colouration characteristic of the presence of a Fe–S centre as a dimer, and the third has a colourless peak as a monomer. Gel filtration analysis of freshly purified recombinant ScGrx6 (Fig. 1a) had a theoretical molecular mass of 23 kDa. The apparent molecular masses of ScGrx6 determined from the upper chromatogram at 280 nm were 46 kDa, 92 kDa, and 184 kDa. The main peaks at the retention volumes of 50 and 70 ml were identified as oligomers (tetramer), dimer and monomers, respectively.

### Grx6 mutagenesis

We created mutations and deletions in the Grx6 sequence to know the effect of them on the Grx6 purification and structure. Proteins with these mutantions and deletions were all successfully expressed as well as purified with a similar yield to the WT protein, with the exception of a mutant with five lysines replaced by alanines in positions 82, 84, 102, 105 and 111, indicating that these residues might be fundamental for the protein folding or stability. Most of the mutations and deletions showed a sharper peak pattern in the purification. We propose some mutations on the loop between the N and C domains significantly interact with the Fe-S and the GSH dependent oxidoreductase activity. To check whether the amino acids between 86 and 101, as well as the active site, are responsible for interaction with Fe-S and the dramatically decreased GSH-dependent oxidoreductase activity of Grx6, we constructed these mutations and deletions and compared them with the wild type. The sequence requiring the assembly of a [2Fe-2S] cluster in Grxs was investigated by expressing proteins with altered active sites. Now, it is clear that the mutant C136A on the active site causes Grx6 to lose the ability to interact with Fe-S and the GSH. In fact, ScGrx6 has one cysteine (Cys) in the sequence, and this Cys is responsible for dimeric but not oligomeric conformations (Fig. 1c). UV-visible absorption studies of samples purified in the presence of GSH showed that Grx6 C136A was no longer able to incorporate a [2Fe–2S]^2+^ cluster. Mutagenesis studies involving cysteine residues of Grx6 were used to investigate the cysteine residues involved in [2Fe–2S]^2+^ cluster ligation. No cluster was observed, indicating that the catalytic cysteine is likely to be a cluster ligand. The higher oligomerization state disappeared by the deletion between 96 and 101 as well as the deletion between 106 and 111 (Fig. 1b & g). It seems that this region is important for the oligomeric state. However, it diminished when the amino acids between 102 and 111 were replaced by 5SA linkers (Fig. 1h). However, when the five lysines were replaced by alanines in positions 82, 84, 102, 105 and 111, the bacteria were not able to express Grx6. We added (NH_4_)_2_Fe(SO_4_)_2_ 6H_2_O to increase the stability by making the protein dimer and tetramer. In addition, we found that the protein concentration increases when we add FeSO_4_·7H_2_O and (NH_4_)_2_Fe(SO_4_)_2_·6H_2_O (Fig. 1k).

Our result shows how the loop between the N-terminal and C-terminal can affect the stability and that it does not depend on the length but depends on the position of amino acid inside the loop. The loop makes significant interactions with other parts of the tetramer, indicating that the region may not be flexible in solution.

### Stability of Fe-S cluster

**Fig. 2:**
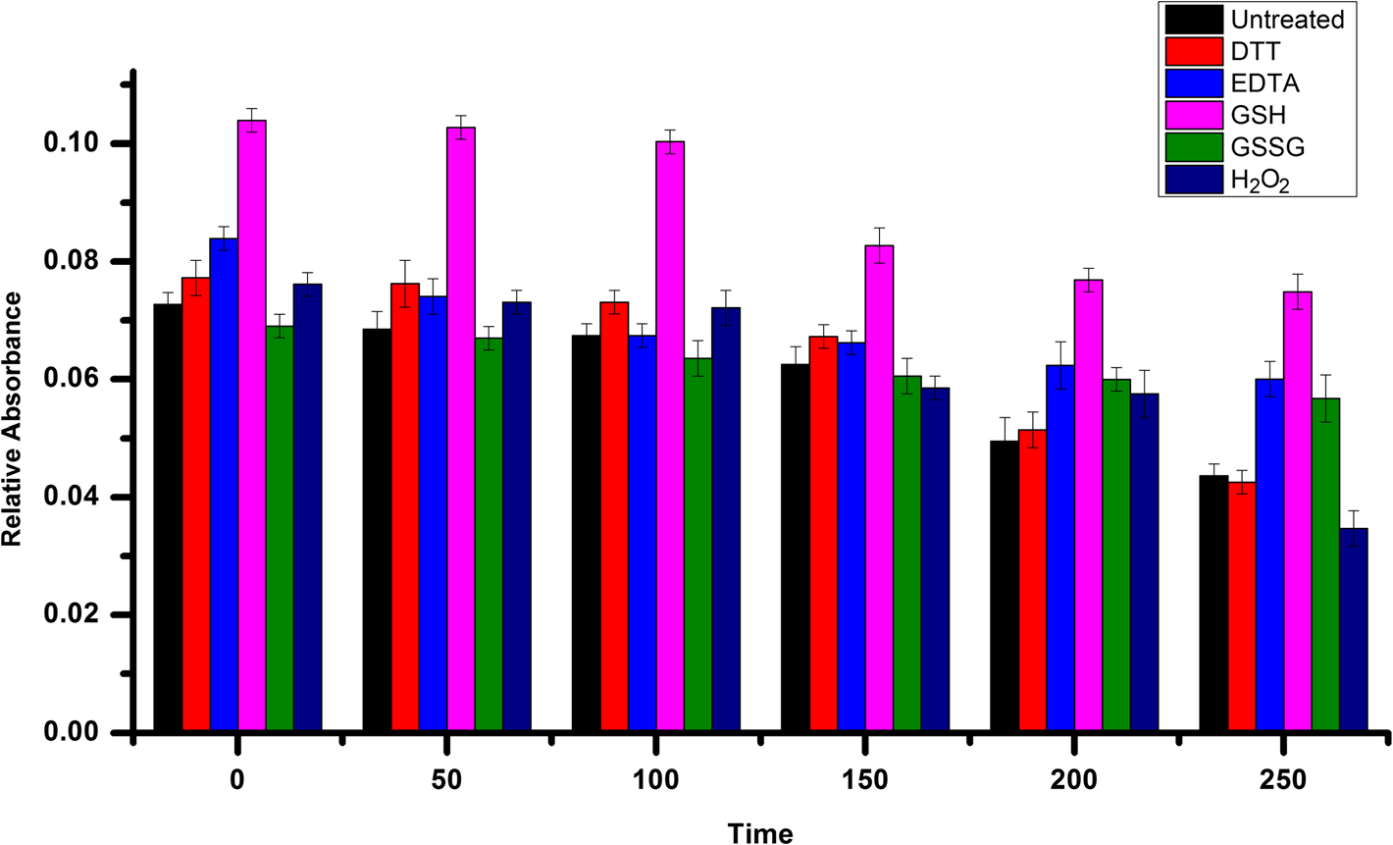
ScGrx6 holoprotein stabilization in aerobic solutions by the addition of DTT, EDTA, GSH, GSSG or H_2_O_2_ at 2 mM. The absorbance of freshly purified ScGrx6 at 430 nm was measured at six different time points in the absence and presence of DTT, EDTA, GSH, GSSG or H_2_O_2_. The absorbance of the buffer containing 20 mM sodium chloride and 50 mM tris hydrochloride, pH 8.0, was subtracted using a reference cuvette. All assays were performed at 25 °C. The concentration of ScGrx6 in the assays was 2 μM. Time points: 0, 50, 100, 150, 200 and 250 min; blank is free of all additives. The experiment was repeated three times

Among the *S. cerevisiae* Grxs, Grx6 was the first one found to form an Fe-S cluster, and this may be a consequence of instability under aerobic conditions. The stability of the Fe-S cluster in the wild-type forms of Grx6 was observed aerobically by measuring the absorption at 430 nm in the presence of different oxidants and reductants as a function of time (Fig. 2). GSH, but not EDTA, GSSG or H_2_O_2_ stabilized the chromophore. However, the stabilizing effect was not solely restricted to the reducing capacity of GSH since ascorbic acid and dithiothreitol had a much weaker effect. In human Grx2, DTT stabilizes the cluster but to a lesser than reduced glutathione.

The cluster in adimeric form in human Grx2 was found in vivo to be inactive in Grxs classical assays [8]. However, the Grx6 dimer form has less activity than the monomer, because of the active side link to the Fe-S. The Fe-S cluster was suggested to work as a redox sensor when the Grx2 is active through conditions of oxidative stress.

### Sequence analysis

The sequence identity between Grx6 and Grx-like domains is in the range 23.76–36.36%. The superposition of the ScGrx6 structure with dithiol/monothiol, reduced/oxidized, GSH bound/unbound and FeS-bound/unbound forms of Grx structures from different organisms shows the root mean square deviation (RMSD) with a range of 1.83–3.96 Å (Table 2), indicating that the overall folding of ScGrx6 is similar to that of other Grxs and Grx-like domains. The sequence identities and the RMSD were calculated by using the PyMOL software [23].

**Table 2:** Comparison of Grx6 with Grxs and Grx-like domains

PDB ID	Protein name	Identity (%)	RMSD(Å)	Organism	References
2cq9	GLRX2 protein	31.73	3.96	*Homo sapiens*	Abe, T et al.(unpublished data)
3d4m	Glutaredoxin-2, mitochondrial	25.96	2.61	*Saccharomyces cerevisiae*	[9]
2fls	Glutaredoxin-2	32.35	2.36	*Homo sapiens*	Johansson et al.(unpublished data)
3d5j	Glutaredoxin-2, mitochondrial	27.18	2.59	*Saccharomyces cerevisiae*	[9]
2e7p	Glutaredoxin	33.00	1.83	*Populustremuloides*	[10]
2ht9	Glutaredoxin-2	31.37	2.68	*Homo sapiens*	[11]
3ctg	Glutaredoxin-2	26.21	2.67	*Saccharomyces cerevisiae*	[12]
1z7r	Glutaredoxin	33.33	2.33	*Populustremuloides*	[13]
3c1s	Glutaredoxin-1	24.04	2.04	*Saccharomyces cerevisiae*	[14]
3rhc	Glutaredoxin-C5, chloroplastic	36.36	2.59	*Arabidopsis thaliana*	[15]
1b4q	Protein (human thioltransferase)	25.74	1.91	*Homo sapiens*	[16]
3h8q	Thioredoxin reductase 3	31.31	2.74	*Homo sapiens*	Chaikuad, A. et al.(unpublished data)
1jhb	Glutaredoxin	23.76	2.21	*Homo sapiens*	[17]
4rqr	Glutaredoxin-1	23.76	2.55	*Homo sapiens*	[18]
3h4k	Thioredoxin glutathione reductase	26.32	2.39	*Schistosomamansoni*	[19]
2x8g	Thioredoxin glutathione reductase	26.32	2.50	*Schistosomamansoni*	[20]
2 × 99	Thioredoxin glutathione reductase	26.32	2.50	*Schistosomamansoni*	[20]
2v6o	Thioredoxin glutathione reductase	26.60	3.08	*Schistosomamansoni*	[21]
3d5j	Glutaredoxin-2, mitochondrial	35.29	2.59	*Saccharomyces cerevisiae*	[9]
2jac	Glutaredoxin-1	30.59	2.06	*Saccharomyces cerevisiae*	[22]
3c1r	Glutaredoxin-1	29.41	2.71	*Saccharomyces cerevisiae*	[14]

**Fig. 3:**
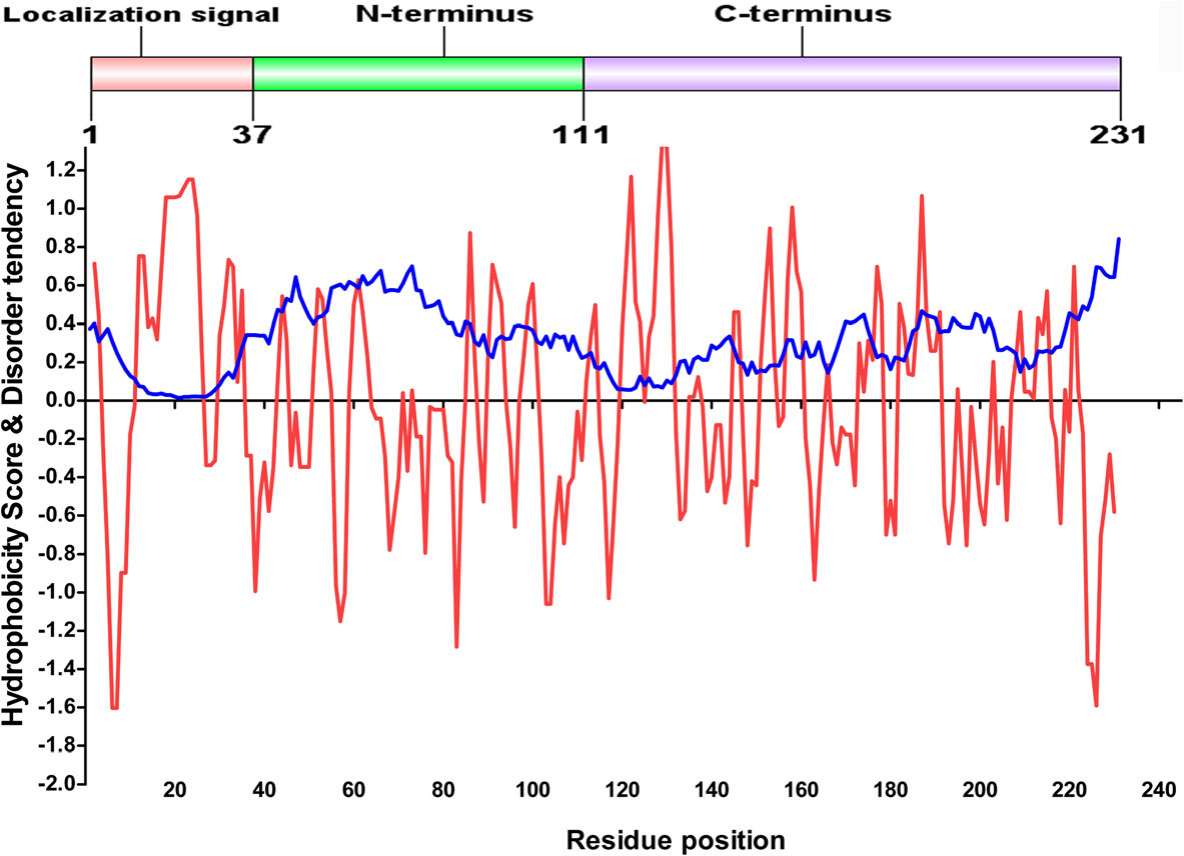
A schematic representation of the ScGrx6 precursor. The regions from left to right are marked in different colours: the signal peptide 37 amino acids, N-domain 74 amino acids and C- domain 120 amino acids. The disorder profile of ScGrx6 sequences is shown underneath. Values above 0.4 are considered disordered (blue). Hydrophilicity and hydrophobicity profiles (> 0 indicates hydrophobicity, while < 0 indicates hydrophilicity) are red. The numbers on the X-axis indicate the position of the residues, and the Y-axis indicates the relative degree of hydrophobicity and disorder. The disorder data available from http://iupred.enzim.hu/pred.php and http://web.expasy.org/protscale/

From these data, we can see the large disordered region from residue 42 through residue 85. It was confirmed that the Grx6 N domain is a slightly hydrophilic peptide based on a hydrophilicity and hydrophobicity analysis. Therefore, it is a prospect that solubility of the Grx6 N domain will be improved with the increase of hydrophilicity of the residues (Fig. 3). It has been demonstrated in the study that the active residue C136 was buried in the hydrophobic region in the dimer form between the two chains of the protein (Additional file 2: Figure S1), while the higher hydrophilic residues were located at the end of the two side of the sequence. The predicted Grx6 sequence contains a cleavage site for all proteolytic enzymes shown in Additional file 3: Table S2. The Grx6 sequence contains antimicrobial domain between 131 and 142 (FSKSTCSYSKGM) Propensity 0.243 Probity 26%.

### Grx6 crystal

**Fig. 4:**
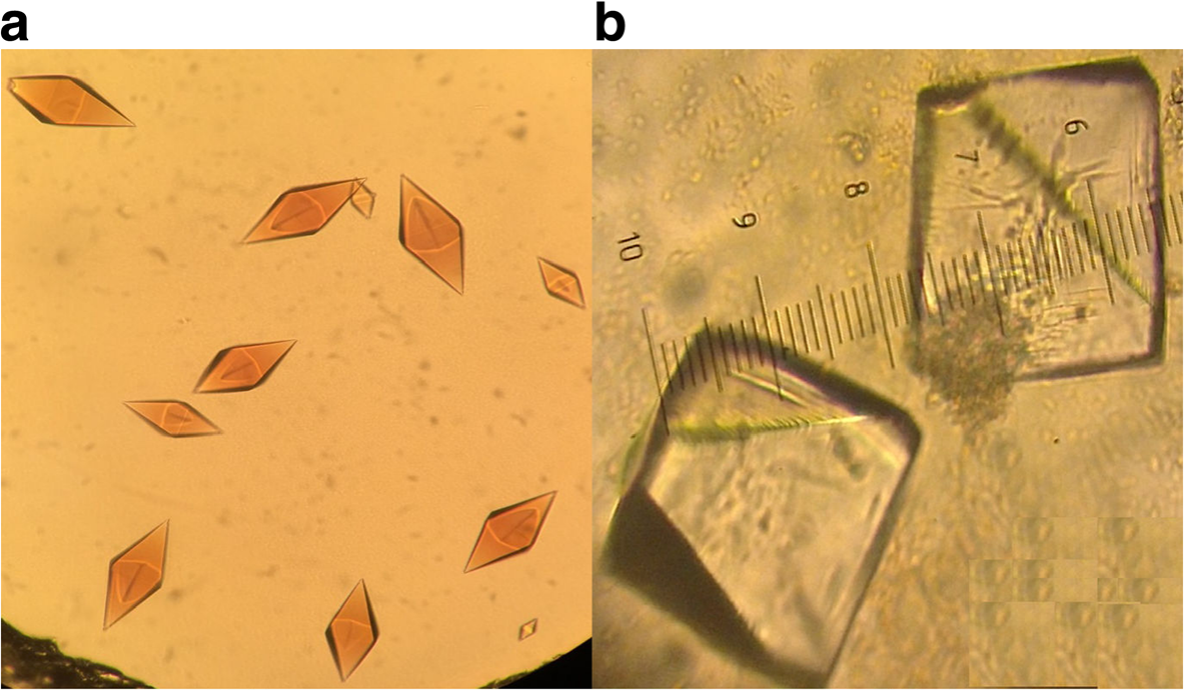
ScGrx6 crystals. Dimer (**a**) and monomer (**b**) crystals

In an earlier work by Abdalla et al. [2], we determined the 3D structure of the reduced holo form of Grx6 by X-ray crystallography. Here, we have shown various differences between dimer and monomer crystals (Fig. 4). Purification chromatography indicated that Grx6 exists as a dimer and monomer in solution, while at native SDS-PAGE analysis dimer and monomer show the same molecular weight. Fe-S increases the stability of many iron-secreted proteins by protecting them against degradation and it may also be required for proper protein folding [24]. However, we realize that the Fe-S cluster may create a difficulty in protein crystallization because of oxidation. The Fe-S cluster conjugated to the secreted proteins may have intrinsic flexibility. In both cases, the dimer and monomer are suitable to protein crystallization. Thus, deletion of the C136 or the C domain increases the flexibility of the protein and complicates crystallization. We subjected the recombinant Grx6 protein to wide crystallization screenings. Reproducible protein crystals were obtained in ~ 35 conditions, and only two crystals were used in the X-ray, and they are stable, meaning that Gx6 crystals have high super saturation, viscosity and solid–liquid interfacial tension. To find full-length crystals or the N-domain crystal, we created all these mutations to circumvent this difficulty. However, all the mutation and deletion proteins are oriented to aggregate in SDS-PAGE analysis and were not suitable for protein crystallization. Both crystals diffracted to a dimer of 2.5 Å and a monomer of 2.1 Å resolution. Surprisingly, we obtained only the C domain structure. To confirm whether these crystals were indeed of full length or the C domain, we isolated one crystal from both and resolved them by SDS–PAGE. Unexpectedly, there is only one band at the full length. We speculated that the N domain helps C domain to make a crystal then aggregates during the stage of C domain crystallization. We speculated that if we can get the N-domain crystal, we will be able to get tetramers in the asymmetric unit of the crystal.

### Protein–ligand interaction

The number of amino acid residues in the monomer and dimer involved for GSH-binding in the active pocket are the same. However, the distance of the hydrogen bonds is slightly longer in the dimer. In the monomer, there are hydrogen bonds between Cys136 and the GSH, while in the dimer, there are ligand bonds between the Cy136 and Fe-S. In addition, there are stronger hydrophobic interactions in the dimer compared to the monomer.

**Fig. 5:**
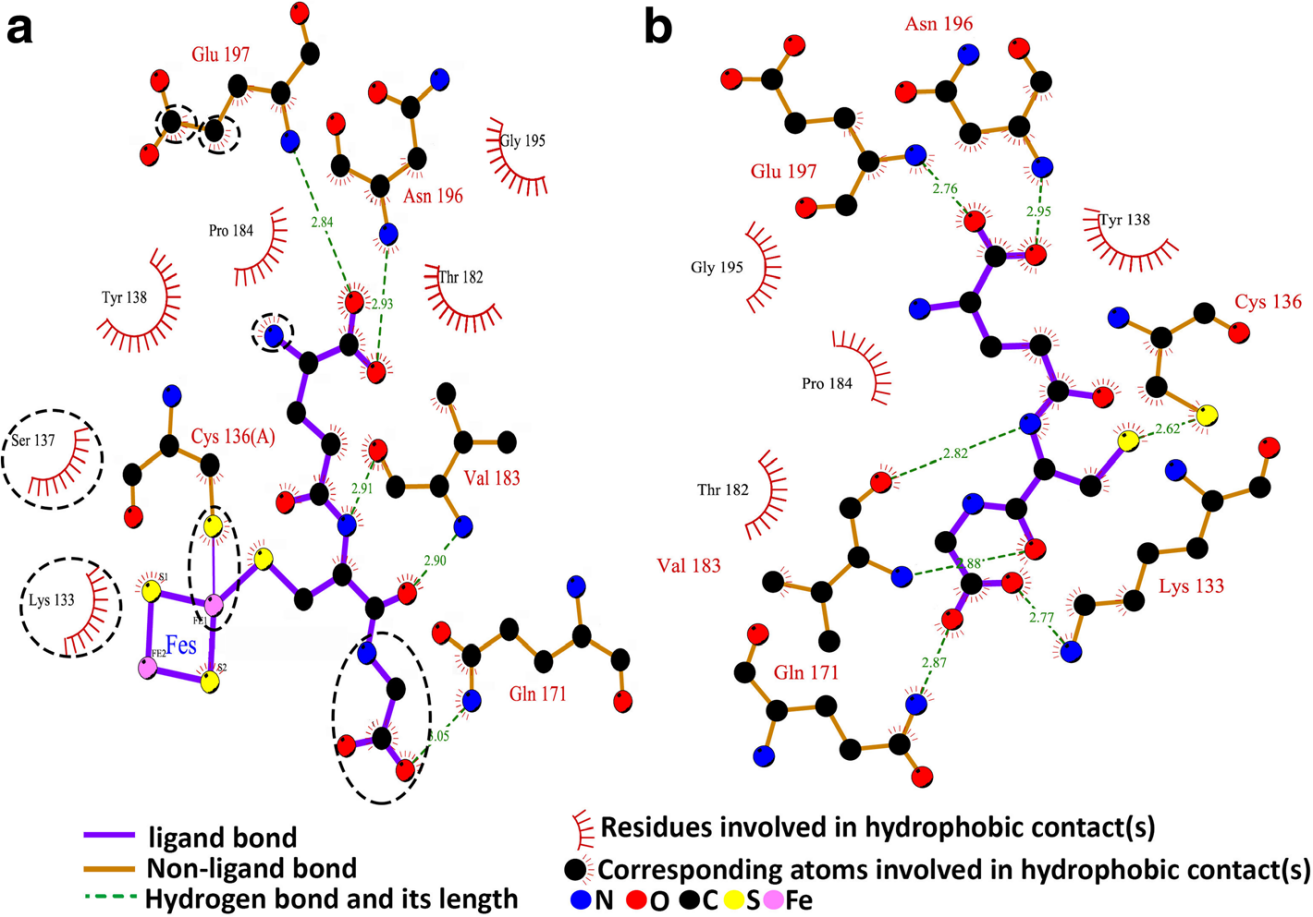
Protein–ligand interaction 2D maps of dimer (**a**) and monomer (**b**); and Fe-S and GSH in the active pocket of ScGrx6. Green dashed lines between the atoms indicate hydrogen bonds with indicated distances (in Å), whereas hydrophobic contacts are shown by an arc with spokes radiating in a direction of the ligand atoms they contact. The contacted atoms are represented with spokes encircled by back dashed lines

There are four amino acid residues (Tyr138, Thr182, Pro184, Gly195) involved in the hydrophobic interaction in the monomer, while there are six amino acid residues (Lys33, Ser137, Tyr138, Thr182, Pro184, Gly195). Additionally, the GSH configuration slightly changes in the dimer when compared to the monomer (Fig. 5, Additional file 4: Figure S2). Grx6 catalyses the chemical reduction of glutathione (GSH) and NADP(+) which forms glutathione disulfide (GSSG), NADPH and FAD (Additional file 5: Figure S3).

### Modelling of the Grx6 N domain

**Fig. 6:**
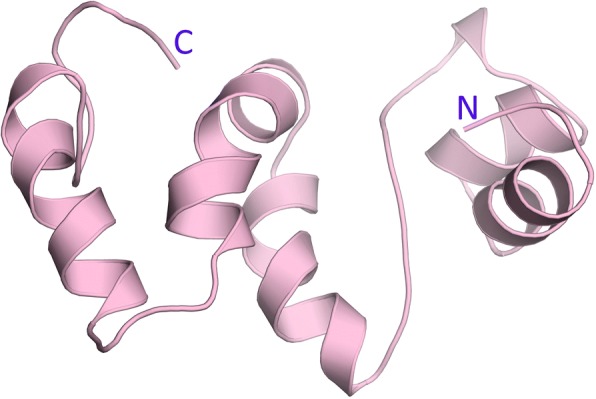
Structure modelling of the ScGrx6 N domain. A homology model was built using Phyre2. Figures were prepared using the PyMOL software (DeLano Scientific, San Francisco, USA)

**Fig. 7:**
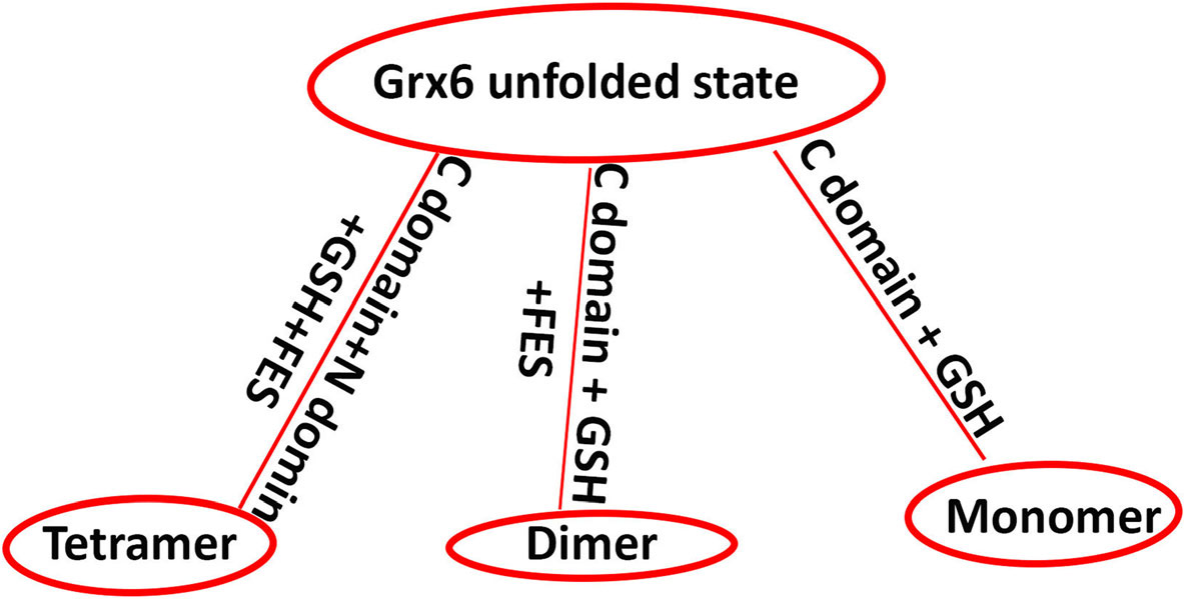
Prediction of ScGrx6 structure

Protein sequences of Grx6 were retrieved from the *Saccharomyces* Genome Database [25]. The amino acid sequence alignment was performed, and BLAST was used to search for the best homology from the Grx6 N domain protein sequences. The hypothetical protein from *Kazachstania africana* shared the largest identity (39%) with the target. The N domain sequence involves 74 amino acids, and 14.7% are Ser with a molecular weight of 8053.85 D and a theoretical pI: 5.02. Moreover, the Grx6 N domain protein contained 12 negatively charged residues and 10 positively charged residues, and had an estimated half-life is 1.3 h and classifies the protein as unstable. The protein-predicted solubility upon overexpression was soluble with a probability of 0.779. The three-dimensional structure shows that Grx6 N domain contains 5 alpha-helices and 6 loops (Fig. 6). The Grx6 N domain has a molecular surface area of 212.091 Å2. We suppose that the N-domain has a role in the formation of Grx6 by making it tetramer (Fig. 7).

### Comparison of the Fe-S cluster positions

**Fig. 8:**
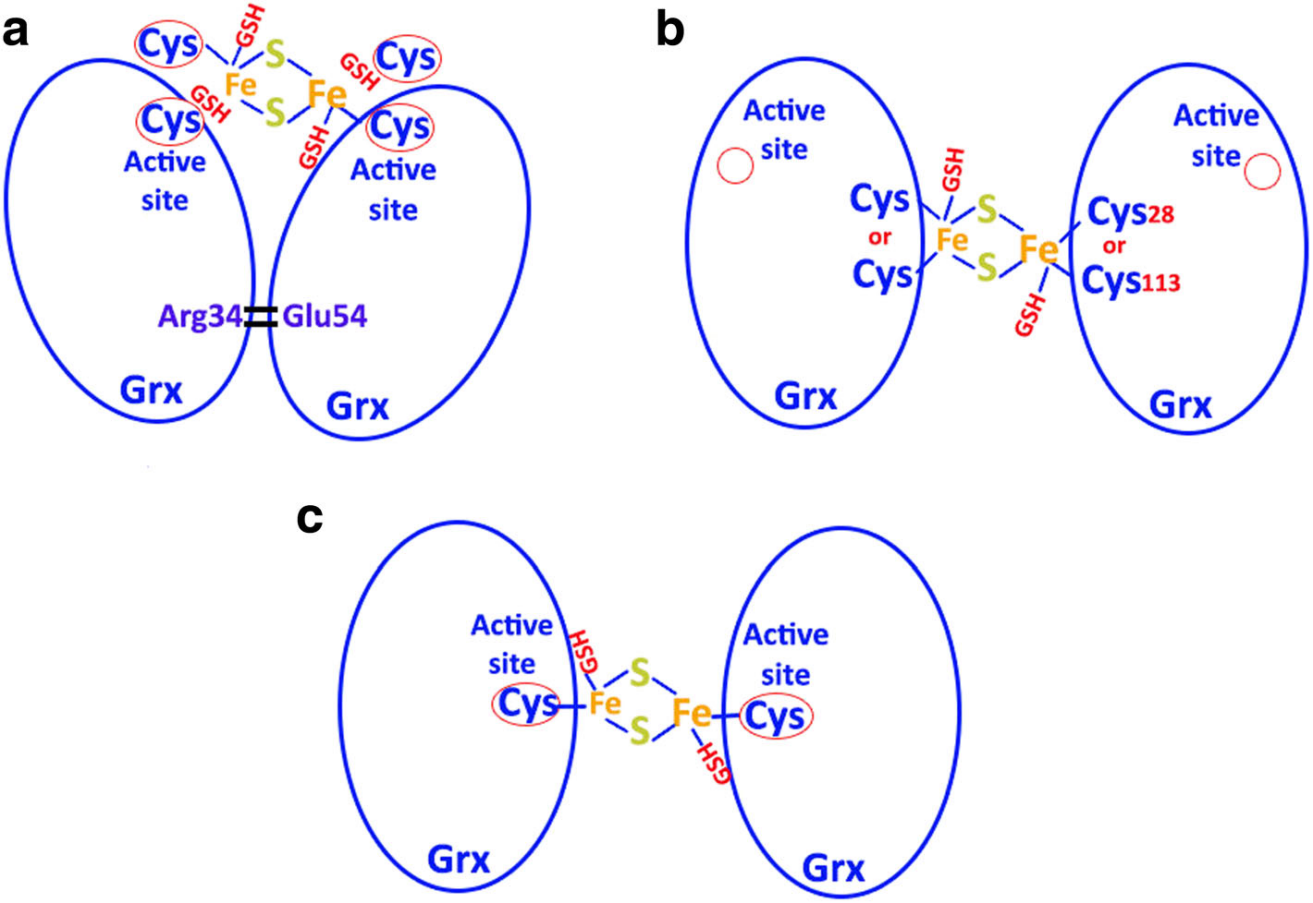
Comparison of the Fe-S cluster positions in (**a**): holo forms of poplar Grx-C1; (**b**), human Grx2; and **c**: ScGrx6

The iron-sulphur clusters (ISCs) in Grxs are bridging two subunits, and both of them are very unique. The ISC in holo Grx-C1 is coordinated via the active site cysteines combined with the cysteines from two GSHs, and the other two GSHs have no hydrogen bond with the Fe-S. Arg34 and Glu54 are responsible for making the tetramer in the presence of Fe-S cluster (Fig. 8a). Human Grx2 contains four cysteines that are divided into two pairs that are relatively close to each other. The first pair consists of Cys-37 and Cys-40, which are reduced and the conserved cysteine-rich motif as implicated in catalysis. The second pair consists of Cys-28 and Cys-113, which form a disulfide bond, and it is on the distal side of the molecule away from the active site cysteines (Fig. 8b). ScGrx6 is different from both of them because it has no hydrogen bonds between Arg and Glu, and it contains only one cysteine in the whole sequence (Fig. 8c).

## Conclusions

In this paper, we used a combination of spectroscopic and proteomic techniques to analyse the sequence and to determine the affected mutations and deletions in the stability of ScGrx6. Our results have increased the knowledge about the differences between monomer and dimer structures in cellular processes and proteins whose roles and functions depend on YCA1 in yeast.

## Additional files

Additional file 1: **Table S1.** Site-directed mutagenesis primers. (DOCX 14 kb)

Additional file 2: **Figure S1.** Hydrophobic interaction between the two subunit of ScGrx6. The distance between two Tyr138 residues is approximately 4.1 Å, which is small enough for their hydrophobic interaction. The hydrophobic interaction between hydrophobic side chains of Tyr138 from both subunits in the interface contributes to the formation of the dimeric interface and helps to stabilize the cluster by reducing the solvent accessibility. (PDF 148 kb)

Additional file 3: **Table S2.** Enzymes cleaving ScGrx6 and the positions of cleavage sites. (DOCX 16 kb)

Additional file 4: **Figure S2.** Ramachandran plot for the predicted model of dimer (A) and monomer (B) of ScGrx6. All residues are in the allowed regions. The Ramachandran plots were performed for quality assessment. Only 120 (52%) of the total 231 residues were present for both dimer and monomer in the disallowed region, whereas no other residues were present in the generously allowed regions (Fig. 2). G-factors provide a measure of how unusual a stereochemical property is. Values below − 1.0 represent high unusualness, while values below − 0.5 represent the unusual property. The G-factors for main chain covalent forces and dihedral angles were calculated to be 0.42 and − 0.44, respectively for the dimer**,** while the G-factors for main chain covalent forces and dihedral angles were calculated to be 0.50 and − 0.41, respectively for the monomer. The overall average G-factor for the dimer was 0.19, and it was 0.28 for the monomer. The Ramachandran plot and G-factors indicate that the backbone dihedral angles, phi, and psi, in the 3D model of dimer and monomer are well within acceptable limits. The Root Mean Square Deviation (RMSD) indicates the degree to which two 3D structures are similar; the lower the value, the more similar the structures. Both template and query structures were superimposed for the calculation of RMSD (Fig. 4). The RMSD value obtained from the superimposition of dimer and monomer using PyMOL view was found to be 0.3 Å over a total of 120 aligned residues. The overall quality factor, Ramachandran plot characteristics, G-factors and RMSD values confirm the quality of the dimer. (PDF 2886 kb)

Additional file 5: **Figure S3.** Grx6 catalyses for the chemical reduction of glutathione (GSH) and NADP(+), which forms glutathione disulfide (GSSG), NADPH and FAD. The Grx6s contain cysteine in their active site making them suitable for oxidation/reduction reactions (Fig. 2). Whereas ScGrx6 can reduce ROS in the cytoplasm, ScGrx6 has the ability to reduce NADP(+) and ScGrx6 catalyses S-glutathionylation to protect SH-groups from oxidation and restore functionally active thiols. ScGrx6 catalyses the reaction only via a monothiol mechanism. Additionally, it has been suggested to have antioxidant capabilities, but their mechanisms are less understood. (PDF 3599 kb)

## Abbreviations

DTT: Dithiothreitol
ER: Endoplasmic reticulum
Fe-S: Iron-sulfur
Grx: Glutaredoxin
GSH: Glutathione
GSSG: Glutathione disulfide
GST: Glutathione-S-tranferease
ISC: Iron-sulphur cluster
PDB: Protein data bank
RMSD: Root mean square deviation
