# Supplementary material for: Structure analysis of yeast glutaredoxin Grx6 protein produced in Escherichia coli
Source: Genes Environ. 2018 Aug 6;40:15. doi: 10.1186/s41021-018-0103-6 (PMC6091153; doi:10.1186/s41021-018-0103-6)
Supplement: Supplementary file 1 — Table S1. Site-directed mutagenesis primers. (DOCX 14 kb) [file 41021_2018_103_MOESM1_ESM.docx]

**Table S1.** Site-directed mutagenesis primers

| A- S | GGGAATTCCATATGAAAGAGGAAACTTCTAAAGCATTTAGT |
| --- | --- |
| A- An | CCGCTCGAGATTATTGGAAGGTTTTTCACGTTGCTC |
| B- S | CTTTATCGTTTTTATCAACCGATGCTATGGGCTG |
| B- An | GCATCGGTTGATAAAAACGATAAAGGGTCGCGAATAAC |
| C- S | GTTCCTTCATGCCCTTGCTATATGAAGCGGTGCTTTTACT |
| C- An | CCGATTATAATATTTAGTAAAAGCACCGCTTCATATAGC |
| D- S | CTCCAACAGCCCATAGCATCGGTTAACGATAAAGGG |
| D- An | GGTTATTCGCGACCCTTTATCGTTAACCGATGCTATG |
| E- S | GAAATAAAACAGAAGGTGGGACTCCAAATAACCAAAGC |
| E- An | CTTTTTGAACATTAAAAGCTTTGGTTATTTGGAGTCCCAC |
| F- S | GAAGGTGGGACTCCAACAGCCCATAGGGTCGCGAATAAC |
| F- An | CATTAAAAGCTTTGGTTATTCGCGACCCTATGGGCTGTTG |
| G- S | CGACCCTTTATCGTTAACCGATGCTATGGGCTGTTGGAGTCC |
| G- An | GCATCGGTTAACGATAAAGGGTCGCGAATAACCAAAG |
| H- S | GATAGTTTGTCGGCCATTAGTGCTAGCGCCAGTGCAAGCGCGAGTGCTGCTTTTAATG |
| H- An | CTTTTTGAACATTAAAAGCAGCACTCGCGCTTGCACTGGCGCTAGCACTAATGGCCGAC |
| I- S | CGACAAACTATCCTGTTGGAGTCCCACCTTCTG |
| I- An | GGACTCCAACAGGATAGTTTGTCGGCCATTAAAAACG |
| J-k82-84 S | GGGCTGTTGGAGTCCCACCGCCTGTGCTATTTCATTAAT |
| J-k82-84 An | AGACGAAGAAATTAATGAAATAGCACAGGCGGTGGGACTCC |
| J-K103K106 S | GGTTATTCGCGACCCTGCATCGTTTGCAATGGCCGAC |
| J-K103K106 An | GATGATAGTTTGTCGGCCATTGCAAACGATGCAGGGTCGCG |
