# Supplementary figures and images for: Structure analysis of yeast glutaredoxin Grx6 protein produced in Escherichia coli
Source: Genes Environ. 2018 Aug 6;40:15. doi: 10.1186/s41021-018-0103-6 (PMC6091153; doi:10.1186/s41021-018-0103-6)

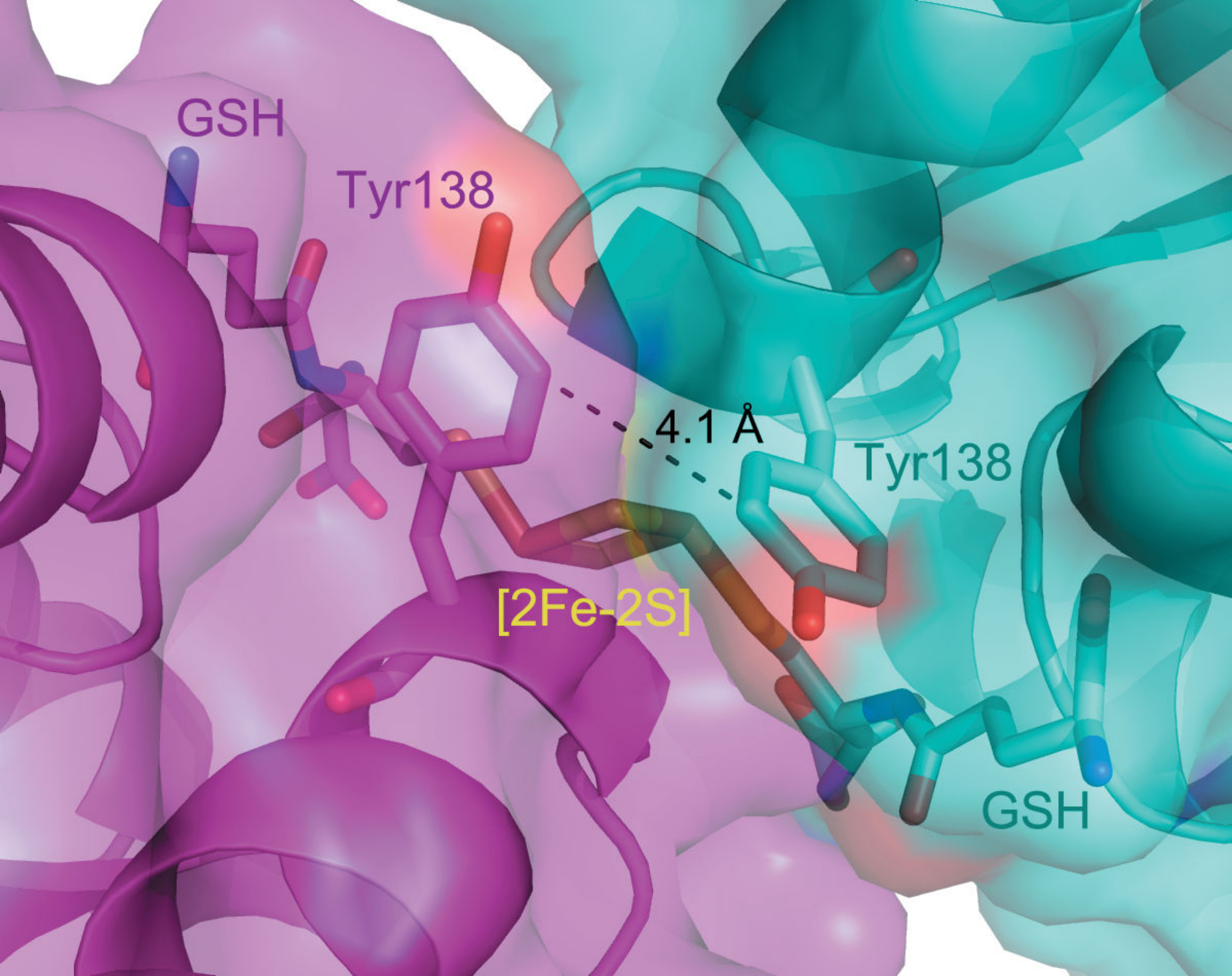

Supplement: Supplementary file 2 — Figure S1. Hydrophobic interaction between the two subunit of ScGrx6. The distance between two Tyr138 residues is approximately 4.1 Å, which is small enough for their hydrophobic interaction. The hydrophobic interaction between hydrophobic side chains of Tyr138 from both subunits in the interface contributes to the formation of the dimeric interface and helps to stabilize the cluster by reducing the solvent accessibility. (PDF 148 kb) [file 41021_2018_103_MOESM2_ESM.pdf]

**A**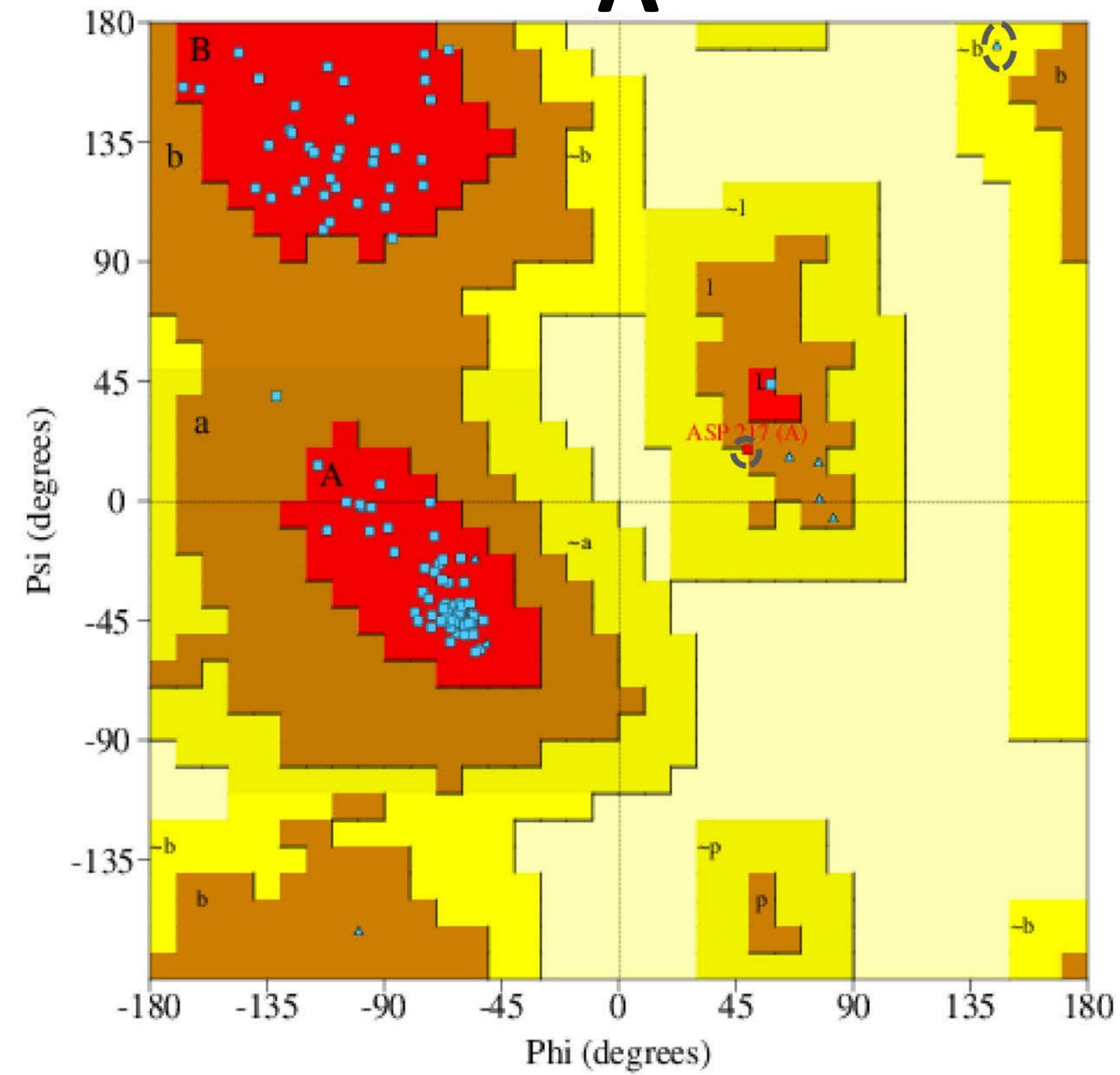**B**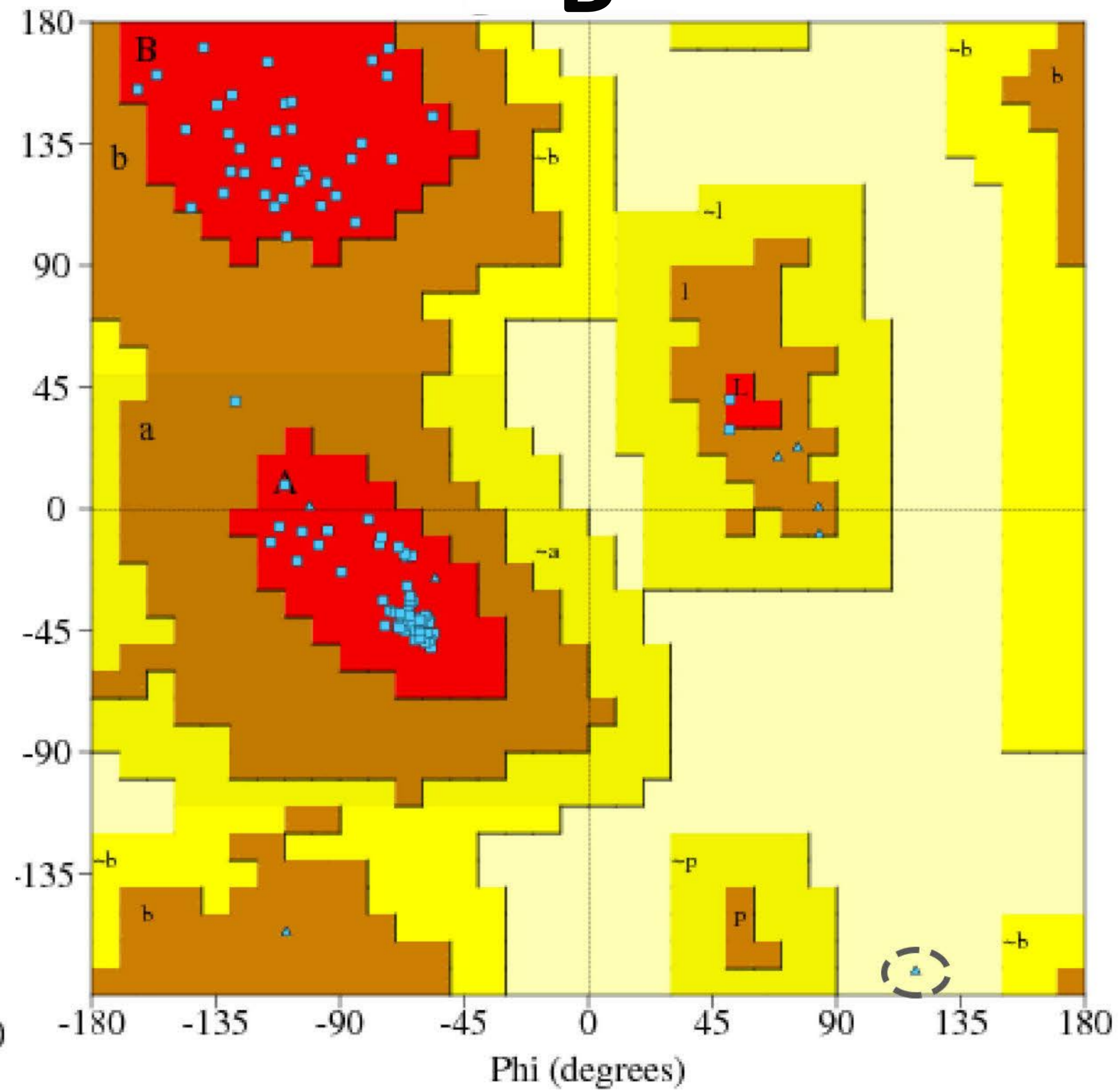

Supplement: Supplementary file 4 — Figure S2. Ramachandran plot for the predicted model of dimer (A) and monomer (B) of ScGrx6. All residues are in the allowed regions. The Ramachandran plots were performed for quality assessment. Only 120 (52%) of the total 231 residues were present for both dimer and monomer in the disallowed region, whereas no other residues were present in the generously allowed regions (Fig. 2). G-factors provide a measure of how unusual a stereochemical property is. Values below − 1.0 represent high unusualness, while values below − 0.5 represent the unusual property. The G-factors for main chain covalent forces and dihedral angles were calculated to be 0.42 and − 0.44, respectively for the dimer, while the G-factors for main chain covalent forces and dihedral angles were calculated to be 0.50 and − 0.41, respectively for the monomer. The overall average G-factor for the dimer was 0.19, and it was 0.28 for the monomer. The Ramachandran plot and G-factors indicate that the backbone dihedral angles, phi, and psi, in the 3D model of dimer and monomer are well within acceptable limits. The Root Mean Square Deviation (RMSD) indicates the degree to which two 3D structures are similar; the lower the value, the more similar the structures. Both template and query structures were superimposed for the calculation of RMSD (Fig. 4). The RMSD value obtained from the superimposition of dimer and monomer using PyMOL view was found to be 0.3 Å over a total of 120 aligned residues. The overall quality factor, Ramachandran plot characteristics, G-factors and RMSD values confirm the quality of the dimer. (PDF 2886 kb) [file 41021_2018_103_MOESM4_ESM.pdf]
