# Supplementary material for: Structure analysis of yeast glutaredoxin Grx6 protein produced in Escherichia coli
Source: Genes Environ. 2018 Aug 6;40:15. doi: 10.1186/s41021-018-0103-6 (PMC6091153; doi:10.1186/s41021-018-0103-6)
Supplement: Supplementary file 3 — Table S2. Enzymes cleaving ScGrx6 and the positions of cleavage sites. (DOCX 16 kb) [file 41021_2018_103_MOESM3_ESM.docx]

|  | No. of cleavages | Positions of cleavage sites |
| --- | --- | --- |
| Arg-C proteinase | 7 | 7 10 57 108 180 193 225 |
| Asp-N endopeptidase | 8 | 48 74 94 95 103 123 161 216 |
| Asp-N endopeptidase + N-terminal Glu | 29 | 34 37 38 48 57 72 74 75 76 79 94 95 103 117 123 143 146 148 159 161 167 168 171 196 197 209 216 222 225 |
| BNPS-Skatole | 1 | 215 |
| CNBr | 5 | 1 48 51 62 142 |
| Chymotrypsin-high specificity (C-term to [FYW], not before P) | 16 | 23 24 31 44 59 113 119 131 138 150 152 156 157 173 215 220 |
| Chymotrypsin-low specificity (C-term to [FYWML], not before P) | 46 | 1 12 17 18 19 20 21 23 24 31 32 44 48 51 59 87 98 113 119 121 123 125 131 138 142 145 146 150 152 156 157 161 164 166 170 173 176 186 187 202 203 208 209 212 215 220 |
| Clostripain | 7 | 7 10 57 108 180 193 225 |
| Formic acid | 8 | 49 75 95 96 104 124 162 217 |
| Glutamyl endopeptidase | 21 | 35 38 39 58 73 76 77 80 118 144 147 149 160 168 169 172 197 198 210 223 226 |
| Hydroxylamine | 1 | 189 |
| Iodosobenzoic acid | 1 | 215 |
| LysC | 20 | 6 37 42 69 82 84 102 105 111 117 133 140 143 163 175 200 201 207 219 227 |
| LysN | 20 | 5 36 41 68 81 83 101 104 110 116 132 139 142 162 174 199 200 206 218 226 |
| NTCB (2-nitro-5-thiocyanobenzoic acid) | 1 | 135 |
| Pepsin (pH 1.3) | 43 | 11 16 17 18 19 20 21 22 23 24 30 31 32 43 87 97 98 112 120 121 122 123 124 130 131 144 146 151 160 161 169 170 175 176 186 187 201 207 208 211 212 219 220 |
| Pepsin (pH>2) | 55 | 11 16 17 18 19 20 21 22 23 24 30 31 32 43 58 87 97 98 112 118 120 121 122 123 124 130 131 137 138 144 146 149 150 151 156 157 160 161 169 170 172 173 175 176 186 187 201 207 208 211 212 214 215 219 220 |
| Proline-endopeptidase[*] | 1 | 228 |
| Proteinase K | 114 | 2 9 11 12 14 15 16 17 18 19 20 21 22 23 24 25 26 29 31 32 33 34 35 36 38 39 40 43 44 46 52 58 59 60 61 64 66 67 73 74 76 77 78 80 81 85 87 91 92 94 98 100 101 109 110 112 113 115 118 119 121 122 123 125 128 129 130 131 135 138 144 145 146 147 149 150 152 153 156 157 158 159 160 161 168 169 170 172 173 174 176 177 178 182 183 186 187 188 191 197 198 199 202 204 208 209 210 212 214 215 220 222 223 226 |
| Staphylococcal peptidase I | 17 | 35 38 58 73 76 80 118 144 147 149 160 168 172 197 210 223 226 |
| Thermolysin | 63 | 8 10 11 13 16 17 18 19 20 21 22 23 24 25 28 30 31 33 42 43 47 50 51 59 60 84 86 90 91 93 97 99 100 108 111 112 114 120 121 122 127 128 129 130 141 145 151 157 158 173 175 176 185 186 187 190 201 207 208 211 213 219 221 |
| Thrombin | 1 | 180 |
| Trypsin | 26 | 6 7 10 37 42 57 69 82 84 102 105 108 111 117 133 140 143 163 175 180 193 200 201 207 219 225 |

**Table S2:** The Grx6 sequence predicted contains a cleavage site for all these enzymes
