# Supplementary material for: Structure analysis of yeast glutaredoxin Grx6 protein produced in Escherichia coli
Source: Genes Environ. 2018 Aug 6;40:15. doi: 10.1186/s41021-018-0103-6 (PMC6091153; doi:10.1186/s41021-018-0103-6)
Supplement: Supplementary file 5 — Figure S3. Grx6 catalyses for the chemical reduction of glutathione (GSH) and NADP(+), which forms glutathione disulfide (GSSG), NADPH and FAD. The Grx6s contain cysteine in their active site making them suitable for oxidation/reduction reactions (Fig. 2). Whereas ScGrx6 can reduce ROS in the cytoplasm, ScGrx6 has the ability to reduce NADP(+) and ScGrx6 catalyses S-glutathionylation to protect SH-groups from oxidation and restore functionally active thiols. ScGrx6 catalyses the reaction only via a monothiol mechanism. Additionally, it has been suggested to have antioxidant capabilities, but their mechanisms are less understood. (PDF 3599 kb) [file 41021_2018_103_MOESM5_ESM.pdf]

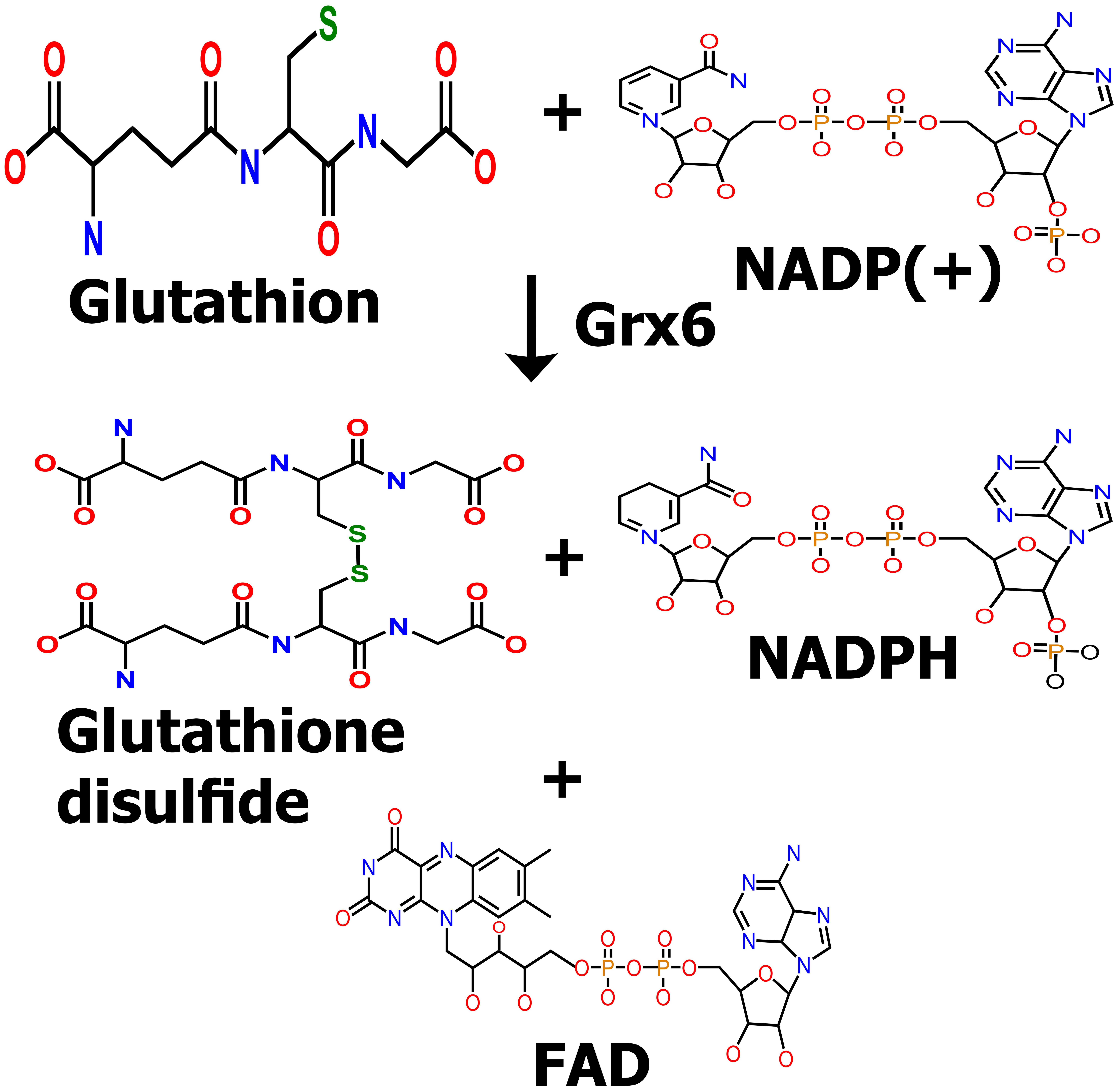
Glutathion
NADP(+)
Grx6
Glutathione disulfide
NADPH
FAD
